# Sarcoid-like Granulomatous Intraocular Inflammation Caused by Vemurafenib Treatment for Metastatic Melanoma

**DOI:** 10.4274/tjo.galenos.2019.79026

**Published:** 2020-03-05

**Authors:** Hilal Eser Öztürk, Yüksel Süllü

**Affiliations:** 1 Ondokuz Mayıs University Faculty of Medicine, Department of Ophthalmology, Samsun, Turkey

**Keywords:** Melanoma, BRAF, vemurafenib, uveitis, sarcoidosis

## Abstract

Vemurafenib is a potent inhibitor of genetically activated BRAF, which is responsible for tumoral proliferation in cutaneous melanoma. A 56-year-old man receiving vemurafenib therapy presented with uveitis. Over the course of the disease, he developed bilateral, granulomatous uveitis with multiple peripheral chorioretinal lesions. Serum angiotensin-converting enzyme levels increased. The patient was diagnosed with probable ocular sarcoidosis related to vemurafenib and was treated with an intravitreal dexamethasone implant. This case is the first report that shows the clinical and angiographic features of a patient with vemurafenib-related sarcoid-like granulomatous uveitis.

## Introduction

Vemurafenib is a potent inhibitor of the BRAF-mitogen-activated protein kinase/extracellular signal-regulated kinase pathway. BRAF mutation is present in almost half of melanoma patients and is responsible for tumoral proliferation in the absence of growth factors. Vemurafenib has been used for the treatment of BRAF mutation-positive late stage (Stage III-C and Stage IV) melanoma since 2011.^1,2^ Vemurafenib-related uveitis has been reported in phase I, II, and III clinical trials, case reports, and case series in the literature.^3,4,5,6,7^ In addition to this, there is an article in the literature that reported 5 patients with sarcoidosis related to vemurafenib therapy for metastatic melanoma.^8^ Sarcoidosis is a multisystem granulomatous disease of unknown etiology. Genetically susceptible individuals may develop an exaggerated immune response to unknown antigens including tumor cells or drugs.^9^ Vemurafenib may stimulate the immune system and then induce sarcoidosis in some patients.

We present here the clinical and angiographic features of a patient with sarcoid-like granulomatous intraocular inflammation which was induced by vemurafenib therapy for metastatic melanoma.

## Case Report

A 56-year-old man with a history of cutaneous melanoma presented with new-onset conjunctival hyperemia and blurred vision in both eyes. The best-corrected visual acuity was 20/30 and intraocular pressure was 10 mmHg in both eyes. Biomicroscopic evaluation revealed fine keratic precipitates, 4+ cells in the anterior chamber and pupillary membrane in both eyes. Fundus examination showed normal findings bilaterally. Staining of the optic disc was detected on fluorescein angiography (FA).

**Figure 1:**
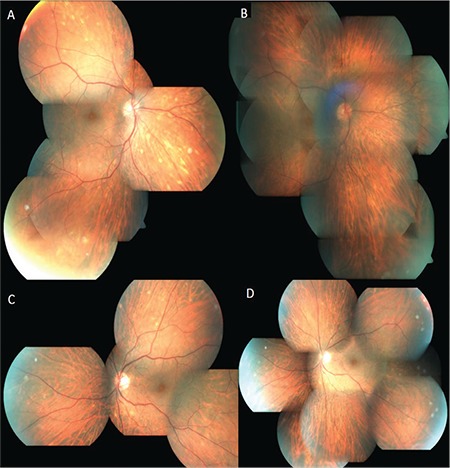
Color fundus photographs show multiple peripheral chorioretinal lesions in the right eye (A, B) and the left eye (C, D), which mostly disappeared within 3 weeks

The patient had been under treatment with vemurafenib 960 mg twice a day for 9 months. Laboratory workup including complete blood count, biochemistry, urine test, and chest X-ray was within normal limits. Serologic tests for infectious diseases including syphilis were negative. Vemurafenib was considered the cause of the uveitis. The oncologist was informed of the situation. However, discontinuation of therapy was not considered because of the life-threatening feature of the disease. Topical corticosteroid and cycloplegic treatment were initiated. During the first week of follow-up, fundus examination revealed multiple peripheral yellow-white lesions that mostly disappeared within 3 weeks (Figure 1).

After 2 months, the patient presented to the clinic because of uveitis recurrence, which had a granulomatous appearance. The patient complained about floaters. His visual acuity was 20/25 in both eyes. Vitreous cells and snowballs were accompanied by a few atrophic chorioretinal lesions. Tuberculin skin test and interferon gamma release assay were negative. Chest computerized tomography was unremarkable. However, serum angiotensin converting enzyme (ACE) level was elevated to 90 U/L (reference range=9-67).

**Figure 2:**
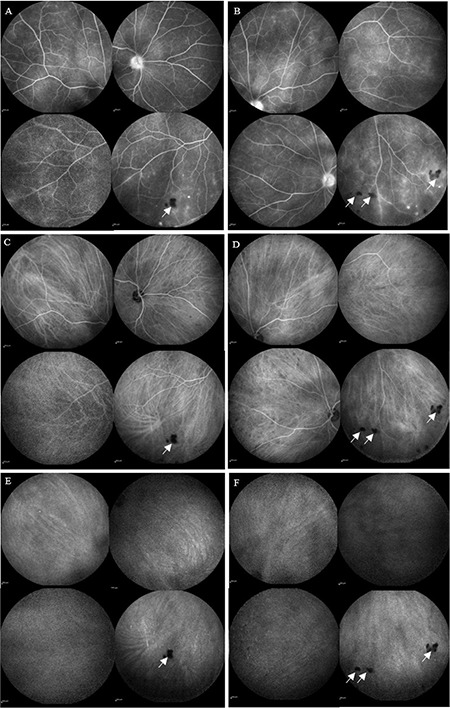
Late-phase fluorescein angiography reveals bilateral staining of the optic disc and vascular leakage in the right eye (A) and left eye (B). Sporadic peripheral hypofluorescent lesions were seen in mid-phase of indocyanine green angiography in the right eye (C) and left eye (D). These lesions disappeared in the late phase in both eyes (E, F). The arrows indicate snowballs

FA showed bilateral staining of the optic disc and vascular leakage. Indocyanine green angiography revealed sporadic peripheral hypo fluorescent lesions that appeared mid-phase and disappeared in the late phase (Figure 2). With these clinical, angiographic, and laboratory results, the patient was diagnosed as having probable ocular sarcoidosis and was treated with intravitreal dexamethasone implant. Intraocular inflammation resolved in a month and has not recurred in 6 months of follow-up. The patient’s visual acuity was 20/25 in both eyes at the final visit. Control FA revealed only late staining of the optic disc bilaterally.

## Discussion

The introduction of vemurafenib and other BRAF inhibitors has been a great improvement in the treatment of advanced cutaneous melanoma. However, they have adverse effects including cutaneous symptoms, arthralgia, nausea, diarrhea, headache, and neutropenia.^1^ Ocular adverse events including uveitis, conjunctivitis, dry eye, episcleritis, and keratitis were also reported with vemurafenib therapy. Uveitis was the most common ocular side effect of vemurafenib in clinical trials.^3^

Lheure et al.^8^ suggested that vemurafenib may induce sarcoidosis or sarcoid-like reactions by increasing serum levels of tumor necrosis factor-a and interferon-g, which induce granuloma formation. They reported 5 patients diagnosed with vemurafenib-related sarcoidosis. Two of the patients had intraocular inflammation. One of them met the criteria for systemic sarcoidosis and the other had Heerfordt syndrome that had been in remission for 15 years and presented with a relapse.^8^ Even though sarcoidosis was not a definitive diagnosis in our patient, bilateral involvement, granulomatous appearance, presence of snowballs, multiple peripheral chorioretinal lesions, negative tuberculin test, and increased serum ACE levels supported probable ocular sarcoidosis according to the international criteria for the diagnosis of ocular sarcoidosis.^10^

Ocular inflammation can usually be controlled by topical, local, and/or systemic corticosteroid therapy in this group of patients. However, treatment guidelines have not been established and management of these patients demands close cooperation with oncologists. Temporary discontinuation of vemurafenib may be suggested to control uveitis. However, some patients need to continue taking the medicine due to the life-threatening nature of the primary disease.^3^ In our case, we cooperated with the patient’s oncologist and decided to treat the patient with local steroids.

Lheure et al.^8^ argued that patients who develop sarcoidosis have a better prognosis with vemurafenib therapy than others. They explained this situation by saying that the activation of the immune system by cytokines may induce both sarcoidal reaction and antitumor response. In our case, cutaneous melanoma has been controlled successfully for 3 years with vemurafenib therapy.

This case is the first report that shows clinical and angiographic features of a patient with vemurafenib-related sarcoid-like granulomatous uveitis and highlights that ocular sarcoidosis should be considered in patients with vemurafenib-related uveitis.
